# Association of Maternal Depression During Pregnancy and Recent Stress With Brain Age Among Adult Offspring

**DOI:** 10.1001/jamanetworkopen.2022.54581

**Published:** 2023-01-30

**Authors:** Klara Mareckova, Radek Mareček, Martin Jani, Lenka Zackova, Lenka Andryskova, Milan Brazdil, Yuliya S. Nikolova

**Affiliations:** 1 Brain and Mind Research, Central European Institute of Technology, Masaryk University, Brno, Czech Republic; 2 1st Department of Neurology, St Anne’s University Hospital and Faculty of Medicine, MU, Brno, Czech Republic; 3 Research Centre for Toxic Compounds in the Environment, Faculty of Science, Masaryk University, Brno, Czech Republic; 4 Campbell Family Mental Health Research Institute, Centre for Addiction and Mental Health, Toronto, Ontario, Canada; 5 Department of Psychiatry, University of Toronto, Toronto, Ontario, Canada

## Abstract

**Importance:**

Maternal mental health problems during pregnancy are associated with altered neurodevelopment in offspring, but the long-term relationship between these prenatal risk factors and offspring brain structure in adulthood remains incompletely understood due to a paucity of longitudinal studies.

**Objective:**

To evaluate the association between exposure to maternal depression in utero and offspring brain age in the third decade of life, and to evaluate recent stressful life events as potential moderators of this association.

**Design, Setting, and Participants:**

This cohort study examined the 30-year follow-up of a Czech prenatal birth cohort with a within-participant design neuroimaging component in young adulthood conducted from 1991 to 2022. Participants from the European Longitudinal Study of Pregnancy and Childhood prenatal birth cohort were recruited for 2 magnetic resonance imaging (MRI) follow-ups, one between ages 23 and 24 years (early 20s) and another between ages 28 and 30 years (late 20s).

**Exposures:**

Maternal depression during pregnancy; stressful life events in the past year experienced by the young adult offspring.

**Main Outcomes and Measures:**

Gap between estimated neuroanatomical vs chronological age at MRI scan (brain age gap estimation [BrainAGE]) calculated once in participants’ early 20s and once in their late 20s, and pace of aging calculated as the differences between BrainAGE at the 2 MRI sessions in young adulthood.

**Results:**

A total of 260 individuals participated in the second neuroimaging follow-up (mean [SD] age, 29.5 [0.6] years; 135 [52%] male); MRI data for both time points and a history of maternal depression were available for 110 participants (mean [SD] age, 29.3 [0.6] years; 56 [51%] male). BrainAGE in participants’ early 20s was correlated with BrainAGE in their late 20s (*r* = 0.7, *P* < .001), and a previously observed association between maternal depression during pregnancy and BrainAGE in their early 20s persisted in their late 20s (adjusted *R^2^* = 0.04; *P* = .04). However, no association emerged between maternal depression during pregnancy and the pace of aging between the 2 MRI sessions. The stability of the associations between maternal depression during pregnancy and BrainAGE was also supported by the lack of interactions with recent stress. In contrast, more recent stress was associated with greater pace of aging between the 2 MRI sessions, independent of maternal depression (adjusted *R^2^* = 0.09; *P* = .01).

**Conclusions and Relevance:**

The findings of this cohort study suggest that maternal depression and recent stress may have independent associations with brain age and the pace of aging, respectively, in young adulthood. Prevention and treatment of depression in pregnant mothers may have long-term implications for offspring brain development.

## Key Points

**Question:**

Is exposure to maternal depression in utero or recent stress associated with offspring brain age in the third decade of life?

**Findings:**

In this cohort study of 260 mother-offspring pairs, greater maternal depression during pregnancy was associated with larger brain age gap in participants in both their early and late 20s, but not with the pace of aging between neuroimaging sessions. In contrast, more recent stress was associated with faster pace of aging between neuroimaging sessions, independent of maternal depression during pregnancy.

**Meaning:**

These results suggest that maternal mental health during pregnancy may have a nonprogressive early association with offspring brain age that remains stable through young adulthood.

## Teaser

This cohort study of Czech mother-offspring pairs examines the association of maternal depression during pregnancy with measures of brain age across multiple follow-ups in offspring in their 20s.

## Introduction

Maternal stress, anxiety, and depression during pregnancy have a long-lasting association with the offspring’s development.^1^ Exposure to maternal anxiety or depression in utero has been associated with a higher risk for emotional and behavioral problems in the child and clinical levels of anxiety and depression in adulthood.^2,3,4,5^ Neuroimaging studies have extended these findings and demonstrated associations between maternal mental health during pregnancy and neurodevelopment in neonates^6,7,8^ as well as young adults.^9,10,11,12,13,14^ Further research showed associations of maternal depression during pregnancy,^12^ exposure to famine in early gestation,^15^ and low birth weight and gestational age^16^ with advanced brain age in the young adult offspring. However, these studies were cross-sectional, and longitudinal research is needed to provide a better understanding of how maternal health during pregnancy might be associated with altered brain age.

Structural brain properties change consistently throughout life.^17^ Therefore, age-dependent changes in brain structure can be used to estimate one’s structural brain age independently of chronological age. Differences between structural and chronological brain age are referred to as the brain age gap estimate (BrainAGE).^18,19^ A greater positive BrainAGE (ie, a structural brain age greater than chronological age) may reflect deviations from normal neurodevelopmental or aging trajectories and has been associated with age-related health problems and neuropsychiatric disease,^20^ as well as cognitive impairment and dementia risk.^21^

Early life adversity has been associated with cross-sectional measures suggestive of possible accelerated brain aging.^12,15^ Franke et al^15^ showed associations between exposure to famine in early gestation and a higher positive BrainAGE in older adult men (ie, late 60s) and research from our group demonstrated correlations between exposure to maternal depression during pregnancy and greater positive BrainAGE in young adults (early 20s) irrespective of sex.^12^ Moreover, the BrainAGE of people in their early 20s showed a quadratic relationship with anxiety and mood dysregulation in young adulthood, suggesting that both delayed as well as accelerated brain maturation may be associated with increased risk for mood and anxiety disorders.^12^ However, these findings were based on a single time point of neuroimaging assessment and thus did not allow us to study whether the observed associations persist or amplify in later life.

Longitudinal data on brain structure can be used to estimate the difference between BrainAGE at different time points, thus providing information regarding the trajectory or pace of aging. However, such longitudinal studies are rare, and so far we are aware of only 1 study calculating the pace of aging using longitudinal data. Critically, this study reported no associations between cross-sectional BrainAGE and the pace of brain aging measured longitudinally.^22^ Instead, it demonstrated that early-life and genetic factors (birth weight and polygenic risk) might have long-lasting relationships with brain structure in adulthood, and that cross-sectional BrainAGE may primarily reflect a preexisting brain condition rather than brain aging.^22^ However, the pace of aging was only investigated in midlife over a period of 2 years. As the authors themselves point out, studies in younger populations may reveal stronger coupling between cross-sectionally assessed BrainAGE and pace of aging, as neurostructural changes occur at a faster rate in earlier life before stabilizing in midlife. Furthermore, this prior study did not consider recent life adversity as a potential moderator of the association between congenital factors and BrainAGE.

According to the diathesis-stress model, a psychological disorder develops as a consequence of an innate or early-life predisposition followed by a set of challenging life events triggering the development of the disorder.^23^ Given strong evidence of a link between early life events and risk factors on brain age,^12,15,22^ early adversity may confer greater vulnerability to accelerated brain aging that may be further amplified by recent stressful life events. Indeed, more stressful life events in midlife were associated with accelerated brain aging.^24^ Similarly, young adult men with posttraumatic stress disorder (PTSD) exhibited a higher positive BrainAGE than healthy controls.^25^ However, to our knowledge no studies to date have applied the diathesis-stress model to brain age.

To address these gaps, we have conducted a second neuroimaging follow-up of the ELSPAC prenatal birth cohort and studied how maternal depression and more recent stress may relate to brain age. Because previous research^26,27^ reported cortical thickness as the best anatomical measure predicting age-related changes, we estimated brain age based on cortical thickness maps. Given the lack of longitudinal data on brain age, we first aimed to assess the associations between BrainAGE at 2 points of participants aged in their 20s and the pace of aging in young adulthood. Next, we aimed to determine whether recent life stress may be a factor in the associations between maternal depression during pregnancy and cross-sectionally assessed BrainAGE or the longitudinally assessed pace of aging. We hypothesized that greater BrainAGE in participants in their early 20s will be associated with greater BrainAGE in their late 20s and a faster pace of aging in young adulthood. Based on the diathesis-stress model, we also hypothesized that recent life stress will moderate the association between maternal depression during pregnancy and BrainAGE in participants’ late 20s and that the greater experiences of recent life stress will be associated with a greater pace of aging in young adulthood.

## Methods

### Participants

A total of 260 young adults between ages 28 and 30 years (52% males, all of White European ancestry) participated in the Health Brain Age study, the second neuroimaging follow-up of the European Longitudinal Study of Pregnancy and Childhood (ELSPAC), a prenatal birth cohort born in the South Moravian Region of the Czech Republic between 1991 and 1992 (eFigure in Supplement 1).^28,29^ Historical data on maternal depression during pregnancy and neuroimaging data from the first neuroimaging follow-up, called Biomarkers and Underlying Mechanisms of Vulnerability to Depression (VULDE), were available for a subset of the participants as detailed below. All participants provided written informed consent to participate in the Health Brain Age study, including the agreement to merge data from Health Brain Age, VULDE, and their historical data from ELSPAC. Ethical approval for the Health Brain Age study was obtained from the ELSPAC ethics committee. This study followed the Strengthening the Reporting of Observational Studies in Epidemiology (STROBE) reporting guideline for cohort studies.

### Procedures

In the early 1990s, mothers of our participants filled in the Edinburgh Postnatal Depression Scale, a self-report questionnaire regarding sadness, anxiety, feeling overwhelmed, crying, sleep difficulties, and self-harming thoughts answered by a 4-point Likert scale. This self-report questionnaire on maternal depression was assessed at 4 time points: twentieth week of pregnancy, 2 weeks after birth, 6 months after birth, and 18 months after birth. In 2015, a subset of the young adult offspring were assessed with structural magnetic resonance imaging (MRI) using a 3T MRI scanner (Siemens Healthineers). From 2020 to 2022, a partially overlapping sample of young adult offspring participated in another MRI of the brain using the same MRI scanner at the Central European Institute of Technology, Masaryk University (CEITEC MU) in the Czech Republic. A total of 131 participants completed the first neuroimaging follow-up (VULDE study) at ages 23 and 24 years, a total of 260 participants completed the second neuroimaging follow-up (Health Brain Age study) at the ages of 28 to 30 years, and a total of 110 participants completed both neuroimaging follow-ups in young adulthood (eFigure in Supplement 1). At the age of 28 to 30 years, all 260 participants also completed the Social Readjustment Rating Scale (SRRS),^30^ a 43-item self-report questionnaire regarding stressful life events in the past year (recent stress), which was used in the subsequent analyses as a continuous variable.

Maternal depression, as well as MRI data at the age of 28 to 30 years, were available for 199 participants (51% males). MRI assessments at both time points (ages 23-24 years and 28-30 years) were available for 110 participants (51% males), allowing to calculate the pace of aging in this subsample. Maternal depression during pregnancy as well as MRI assessments at both time points were available for 87 participants (51% males).

### Calculation of Brain Age and the Pace of Aging

The brain age calculation was conducted as described in Mareckova et al.^12^ Briefly, T1-weighted data were processed using FreeSurfer version 7.1.1,^31^ and the outputs were visually inspected for common artifacts (eg, skull strip failure, spikes, parcellation issues, faulty gray and white matter boundaries). All participants passed these quality control procedures. Next, the Neuroanatomical Age Prediction using R (NAPR)^32^ platform was used to calculate participants’ brain age. The NAPR platform is a cloud-based tool (Amazon Web Services) that estimates the age of an individual using cortical thickness maps derived from their own locally processed T1-weighted whole-brain MRI scans.^32^ This age estimation model was trained on data from 2367 healthy control participants from ages 6 to 89 years using relevance vector machine regression^33^ and Gaussian processes machine learning methods^34^ applied to cortical thickness surfaces obtained using FreeSurfer. Finally, BrainAGE was calculated as the difference between this cortical thickness-based estimate of brain age and each participant’s chronological age. The pace of aging during the 5 years was calculated as the difference between the BrainAGE of participants aged 28 to 30 years (hereafter late 20s) using Health Brain Age assessments and the results of participants between ages 23 and 24 years (early 20s) using VULDE assessments.

### Statistical Analyses

All statistical analyses were performed in JMP version 10.0.0 (SAS Institute Inc). First, we assessed the correlations among BrainAGE (calculated as brain age − chronological age) of participants in their early 20s, BrainAGE in their late 20s, and the pace of aging during the 5 years between the 2 MRI sessions. Next, we used *t* tests to assess sex differences in BrainAGE at each time point and the pace of aging between time points. Finally, we used linear regressions to assess the association between recent stress (continuous variable) and the BrainAGE in the late 20s cohort, as well as the pace of aging during the 5 years. Sex was used as a covariate. Race and ethnicity were not analyzed as a covariate because all participants were of White race and European ethnicity.

Subsequent analyses sought to replicate the Mareckova et al^12^ findings regarding the association between maternal depression during pregnancy and BrainAGE in adults in their early 20s (VULDE study) in a partially overlapping sample of participants approximately 5 years older (Health Brain Age study). A linear mixed model in the 87 participants with historical data on maternal depression during pregnancy as well as both MRI time points assessed the potential interaction between maternal depression during pregnancy and time of MRI assessment on BrainAGE. This model allowed us to analyze changes in BrainAGE over time, taking into account the within-participant correlations between BrainAGE scores (random slope and random intercept per individual is introduced in the model).

Because there was no interaction with the time of MRI assessment, further analyses assessed the association between maternal depression during pregnancy and the BrainAGE in the full Health Brain Age sample with both maternal depression and MRI data in participants’ late 20s (197 participants). Sex was used as a covariate and the potential moderating role of recent stress was tested using the full factorial general linear model. Similarly, we also assessed the association between maternal depression during pregnancy, sex, and recent stress on the pace of aging in young adulthood.

As a control analysis evaluating the potential role of postnatal maternal depression or general anxiety and mood dysregulation in the young adult offspring, we also tested the associations between (1) a perinatal maternal depression factor (eTables 3 and 4 in Supplement 1), BrainAGE of participants in their late 20s, and the pace of aging, and (2) BrainAGE in participants in their late 20s, the pace of aging, and a general anxiety and mood dysregulation factor score (eTable 5 in Supplement 1).

## Results

### Demographic Information

The whole Health Brain Age sample had a mean (SD) age of 29.5 (0.6) years (135 [52%] male); the subsample with both Health Brain Age and VULDE data had a mean age of 29.3 (0.6) years (56 [51%] male) (eTable 1 in Supplement 1). Information on medication during pregnancy is provided in eTable 2 in Supplement 1.

### BrainAGE and the Pace of Aging in Young Adulthood

**Figure 1.**
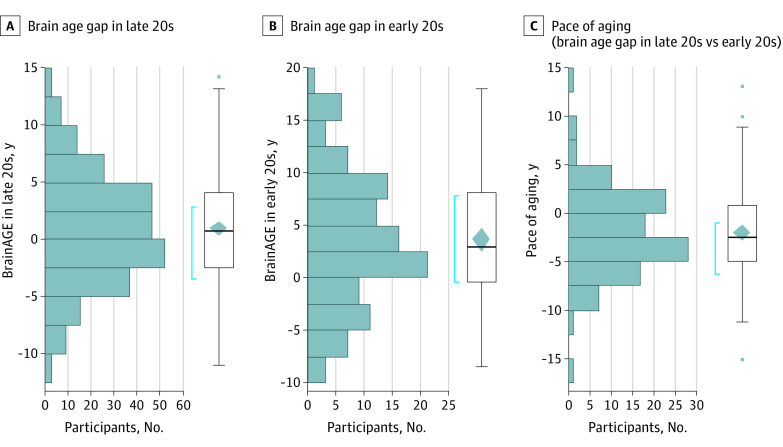
Brain Age Gap Estimate (BrainAGE) in the Early and the Late 20s and the Pace of Aging Over 5 Years Between 2 Magnetic Resonance Imaging Assessments A, The brain age gap in participants’ late 20s varied from −11 to 14 (mean [SD] BrainAGE, 1.0 [4.8]). B, The brain age gap in participants’ early 20s varied from −8 to 18 (mean [SD] BrainAGE, 3.6 [6.2]). C, The pace of aging in young adulthood varied from −15.19 to 13.02 (mean [SD], −1.93 [4.45]).

**Figure 2.**
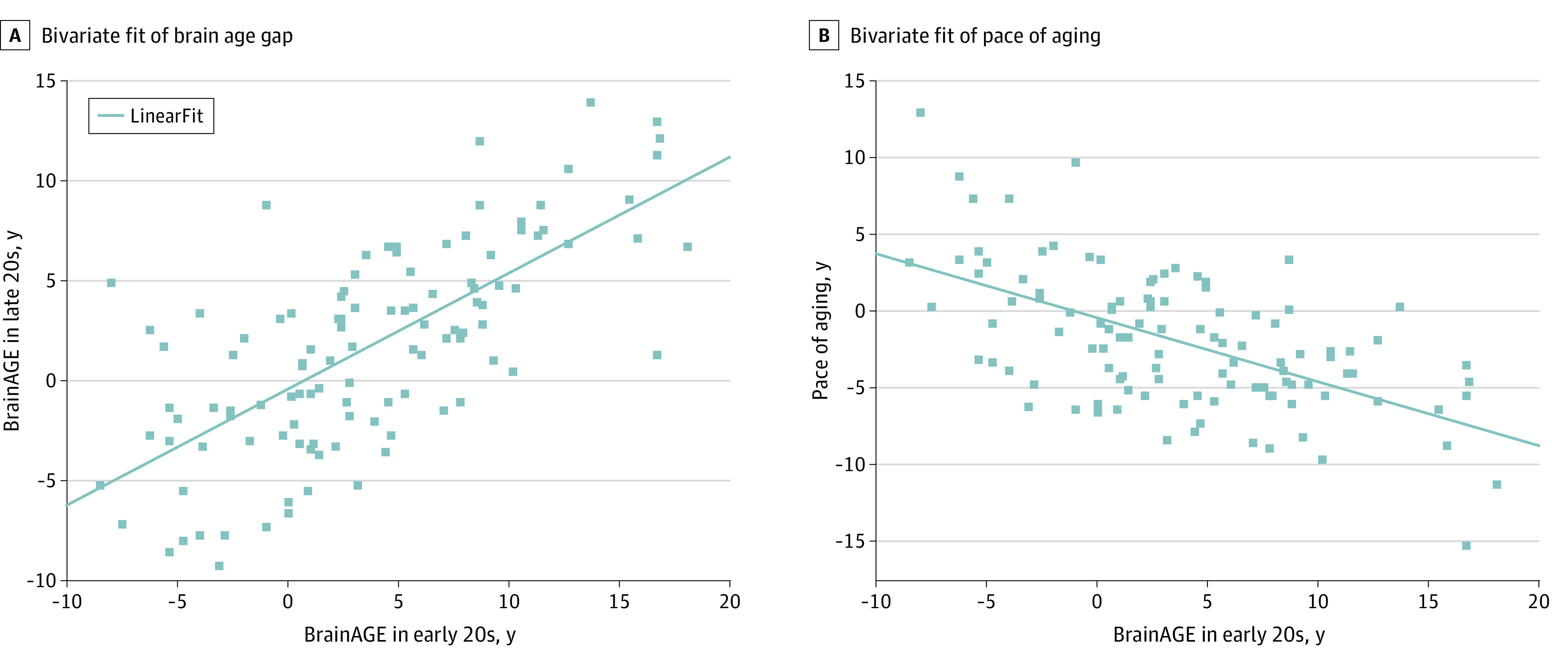
Correlations Between the Brain Age Gap Estimate (BrainAGE) in Participants’ Early and Late 20s, and the Pace of Aging Over the 5 Years Between the 2 Magnetic Resonance Imaging Assessments A, There was a high correlation between the brain age gap in participants’ early 20s and late 20s (*r* = 0.7, *P* < .001). B, There was a high correlation in the pace of aging in young adulthood (*r* = −0.58, *P* < .001).

All 260 participants of the Health Brain Age study were between ages 28 and 30 years, but their brain age varied from 18.5 to 43.3 years (mean [SD] brain age, 30.5 [4.9] years). Their BrainAGE in their late 20s, calculated as the difference between brain age and chronological age, thus varied from −11 to 14 (mean [SD], 1.0 [4.8]) (Figure 1A). A similar range of BrainAGE was observed in the subset of these Health Brain Age participants who also took part in the VULDE study in their early 20s (110 participants; range, −8 to 18; mean [SD], 3.6 [6.2]) (Figure 1B). The correlation between the BrainAGE of participants in their early 20s and late 20s was high (110 participants; *r* = 0.7, *P* < .001) (Figure 2A). The pace of aging in young adulthood, calculated as the difference between the BrainAGE in participants’ late 20s minus the BrainAGE in their early 20s, varied from −15.19 to 13.02 (mean [SD], −1.93 [4.45]) (Figure 1C). Thus, while some participants experienced an acceleration of their pace of aging within the 5 years between the Health Brain Age and VULDE assessments, others experienced a deceleration. Intriguingly, BrainAGE in participants’ early 20s was highly correlated with the pace of aging in the next 5 years (110 participants; *r* = −0.58, *P* < .001) (Figure 2B). A greater positive BrainAGE in participants’ early 20s was associated with a slower pace of aging in the subsequent 5 years.

### Sex Differences in the Brain Age Gap and the Pace of Aging in Young Adulthood

While no sex differences were observed for the BrainAGE of 110 participants in their early 20s, a small sex difference in BrainAGE emerged in their late 20s (260 participants; Cohen *d* = 0.32, *P* = .01). Women had a more positive BrainAGE than men (mean [SD], 1.8 [4.9] vs 0.2 [4.7]). There were no significant sex differences in the pace of aging over the 5 years (110 participants).

### Recent Stress and Its Association With BrainAGE and the Pace of Aging in Young Adulthood

The association between the exposure to recent stress (continuous variable) and the general anxiety and dysregulated mood was also observable in our data (260 participants; β = 0.22, *P* < .001, *R^2^* = 0.05) (eResults in Supplement 1). While there was no association between recent stress and BrainAGE in participants’ late 20s (260 participants; β = 0.03, *P* = .63), greater recent stress was associated with greater pace of aging in young adulthood (110 participants; β = 0.20, *P* = .03, adjusted *R^2^* = 0.05).

### Maternal Depression During Pregnancy and Its Association With BrainAGE and the Pace of Aging in Young Adulthood

Based on the cutoff score for depression measured by Edinburgh Postnatal Depression Scale,^35^ 33% of mothers scored 13 or higher, thus indicating symptomatology of antenatal depression. A linear mixed model showed an association between maternal depression during pregnancy and BrainAGE (87 participants; *F* statistic, 6.07; *P* = .02) and there was no interaction with the time of MRI assessment (*F* statistic, 1.06; *P* = .36). Moreover, the effect size of maternal depression during pregnancy was larger (*F* statistic, 7.27; *P* = .01) when correcting the mixed model for offspring sex and the potential interaction between maternal depression during pregnancy and offspring sex. Given the lack of interaction with the time of MRI assessment, further analyses assessed the correlations between maternal depression during pregnancy, sex, recent stress, and BrainAGE only in the larger sample of HBA participants.

**Figure 3.**
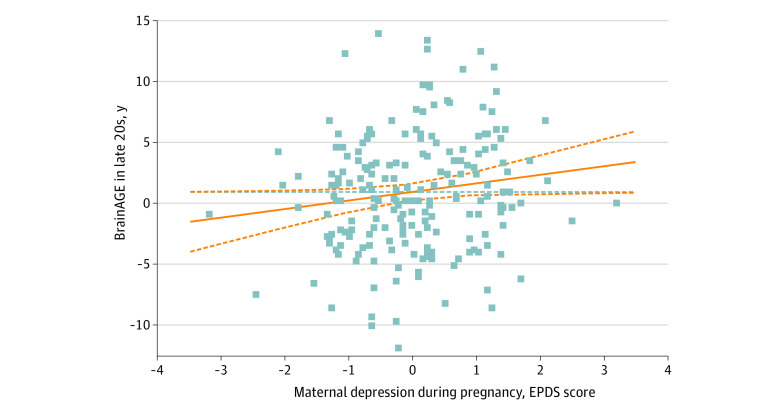
Greater Maternal Depression During Pregnancy and Brain Age Gap Estimate (BrainAGE) in Participants’ Late 20s EPDS indicates Edinburgh Postnatal Depression Scale. This association was independent of sex, stressful life events in the past year, or interaction between maternal depression during pregnancy and stressful life events in the past year (β = 0.14, *P* = .04, adjusted *R^2^* = 0.04).

**Figure 4.**
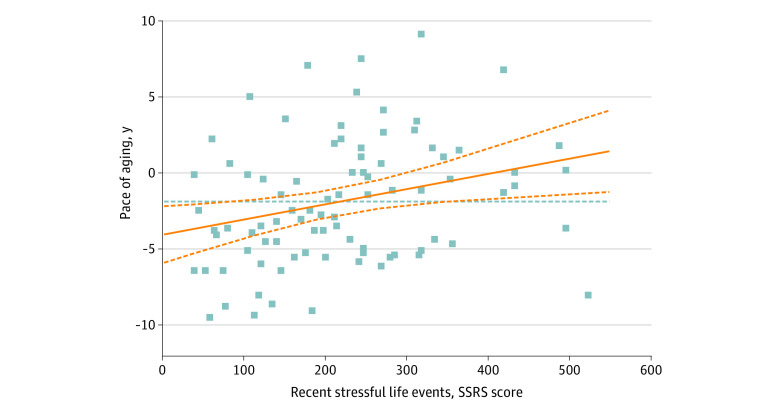
Stress in the Past Year and Pace of Aging in Young Adulthood SSRS indicates Social Readjustment Rating Scale. The effect size of the association between stress in the past year and pace of aging was greater after correcting for sex, maternal depression during pregnancy, and the possible interaction between maternal depression during pregnancy and recent stress (β = 0.028, *P* = .01, adjusted *R^2^* = 0.09).

Greater maternal depression during pregnancy was associated with greater positive BrainAGE in participants’ late 20s independently of sex, recent stress, or interaction between maternal depression during pregnancy and recent stress (199 participants; β = 0.14, *P* = .04, adjusted *R^2^* = 0.04) (Figure 3). This model showed that there was a association with sex (β = 0.17, *P* = .01) but no association with recent stress (β = 0.03, *P* = .72) or any interaction between maternal depression during pregnancy and recent stress (β = 0.01, *P* = .87). A similar model focusing on the pace of aging in young adulthood showed an association with recent stress (110 participants; β = 0.028, *P* = .01, adjusted *R^2^* = 0.09) (Figure 4) but no association with maternal depression during pregnancy (β = −0.19, *P* = .08), sex (β = 0, *P* = .09), or any interactions between maternal depression during pregnancy and recent stress (β = 0.19, *P* = .08) on the pace of aging in young adulthood. There was no association between any of the brain age metrics and perinatal maternal depression or general anxiety and mood dysregulation in the late 20s (eTable 5 in Supplement 1).

## Discussion

In this cohort study, we conducted a second neuroimaging follow-up of the ELSPAC prenatal birth cohort in young adulthood, demonstrated that a previously reported association between maternal depression during pregnancy and BrainAGE of offspring in their early 20s was replicated in their late 20s, and showed that these associations were independent of sex, recent stress, or any interaction with recent stress. This lack of interaction and the high correlation (*r* = 0.7) between BrainAGE in participants’ early 20s and their late 20s suggest that the associations between exposure to maternal depression in utero and BrainAGE are stable in the third decade of life and do not support a diathesis-stress model linking maternal depression and offspring brain age. We conclude that deviations in brain age may be partially programmed prenatally and that maternal depression during pregnancy may have a long-lasting nonprogressive relationship with brain age of the offspring.

Our findings are in line with those of Franke et al,^15^ who showed an association between maternal nutrient restriction due to famine in early gestation and a greater positive brain age gap measured cross-sectionally in late adulthood. They are also consistent with those of Vidal-Pineiro et al^22^ and Hedderich et al,^16^ who showed an association between lower birth weight and greater positive BrainAGE measured cross-sectionally. Together, these studies point out that critical lifelong relationships with brain structure may be programmed in early life.

While recent stress was not associated with BrainAGE in participants’ late 20s, exposure to recent stress was associated with the pace of aging in young adulthood, as well as general anxiety and dysregulated mood, and these findings were independent of sex. The directionality of these findings is consistent with Hatton et al,^24^ who reported an association between stressful life events in midlife and a greater positive BrainAGE, as well as an ENIGMA (Enhancing NeuroImaging Genetics through Meta Analysis) study on PTSD,^25^ which reported greater positive BrainAGE in young adult men with PTSD vs healthy controls. However, both of these studies measured the BrainAGE only cross-sectionally.

BrainAGE measured in participants’ early 20s highly correlated with both BrainAGE in their late 20s as well as the pace of aging in young adulthood. However, contrary to our expectations, a greater positive BrainAGE in participants’ early 20s was associated with a slower pace of aging in the subsequent 5 years, suggesting that those with more advanced brain development at the ages of 23 and 24 years may have reached a plateau and thus their subsequent pace of aging was slower. This possible interpretation is in line with Fjell et al,^36^ who studied changes in cortical thickness over the lifespan and reported a high rate of cortical thinning for the first 20 years of life followed by a steadier rate of thinning. Consistent with research on the steeper thickness decline in women vs men until the age of 40,^37^ we also found a slightly higher positive BrainAGE in women vs men in their late 20s.

### Limitations

Our study has several limitations. First, the sample size was relatively small, especially for the pace of aging analyses in the subsample with both MRI assessments in young adulthood (110 participants). This subsample would allow us to detect correlations between maternal depression during pregnancy and pace of aging of *r* = 0.26 or higher, with a power of 80% and *P* = .05. Future research should verify our findings in a larger sample and test their stability across different developmental periods (eg, childhood). Second, our study used a cortical thickness-based estimator of brain age but there are also other structural as well as functional measures of the brain, which could be used for brain age measurement. Future research might verify the associations between maternal depression during pregnancy, recent stress, and brain age based on these different metrics. Third, the Social Readjustment Rating Scale does assess stressful life events in the past year but does not give an indication of when the events occurred. While previous research reported significant changes in brain structure between 2 phases of menstrual cycle^38^ as well as after a 7-day training in juggling,^39^ the most recent events (eg, happening immediately before the MRI assessment) likely did not influence the neuroanatomy and thus brain age, which might have added noise to our data and reduced our power to detect the interaction between maternal depression during pregnancy and recent stress. Finally, while we did not find any associations between postnatal maternal depression and brain age, suggesting that genetic liability to depression is not driving our findings, we cannot exclude the possibility that variables correlated with maternal depression during pregnancy are contributing to our findings.

## Conclusions

In this cohort study, the brains of the offspring exposed to maternal depression in utero appeared older than their chronological age, and these associations were present in participants’ early 20s as well as their late 20s. The stability of the association between maternal depression during pregnancy and BrainAGE was also supported by the lack of any interactions with recent stress and the lack of a significant association between maternal depression during pregnancy and the pace of aging between individuals’ early and late 20s. These findings support the existence of long-lasting relationships between maternal mental health during pregnancy and offspring brain development and point out the importance of targeting possible interventions at the pregnant mother in addition to the affected offspring.

## Notes

Supplement 1. **eFigure.** Recruitment Flow Diagram and Sample Size of the Different Analyses **eTable 1.** Demographics Table **eTable 2.** Medication During Pregnancy **eTable 3.** Correlation Matrix Describing the Relationships Between Maternal Depression Measured During the Early Life of the Offspring **eTable 4.** Loadings of the Main Factor Describing Maternal Depression During the Early Life of the Offspring **eTable 5.** Loadings of the Main Factor Describing Anxiety and Dysregulated Mood in Offspring in Their Late 20s
eResults.

eReferences.

Supplement 2.
Data Sharing Statement
