# Supplementary material for: Association of Maternal Depression During Pregnancy and Recent Stress With Brain Age Among Adult Offspring
Source: JAMA Netw Open. 2023 Jan 30;6(1):e2254581. doi: 10.1001/jamanetworkopen.2022.54581 (PMC9887495; doi:10.1001/jamanetworkopen.2022.54581)
Supplement: Supplement 1. — eFigure. Recruitment Flow Diagram and Sample Size of the Different Analyses eTable 1. Demographics Table eTable 2. Medication During Pregnancy eTable 3. Correlation Matrix Describing the Relationships Between Maternal Depression Measured During the Early Life of the Offspring eTable 4. Loadings of the Main Factor Describing Maternal Depression During the Early Life of the Offspring eTable 5. Loadings of the Main Factor Describing Anxiety and Dysregulated Mood in Offspring in Their Late 20s eResults. eReferences. [file jamanetwopen-e2254581-s001.pdf]

Supplemental Online Content

Mareckova K, Marecek R, Jani M, et al. Association of maternal depression during pregnancy and recent stress with brain age among adult offspring. *JAMA Netw Open.* 2023;6(1):e2254581. doi:10.1001/jamanetworkopen.2022.54581

eFigure. Recruitment Flow Diagram and Sample Size of the Different Analyses

eTable 1. Demographics Table

eTable 2. Medication During Pregnancy

eTable 3. Correlation Matrix Describing the Relationships Between Maternal Depression Measured During the Early Life of the Offspring

eTable 4. Loadings of the Main Factor Describing Maternal Depression During the Early Life of the Offspring

eTable 5. Loadings of the Main Factor Describing Anxiety and Dysregulated Mood in Offspring in Their Late 20s

eReferences.

This supplemental material has been provided by the authors to give readers additional information about their work.

eFigure. Recruitment flow diagram and sample size of the different analyses. The second neuroimaging follow-up of the ELSPAC prenatal birth cohort (Health Brain Age study) was done in 260 young adults (age 28-30), who were chosen from the large cohort based on a first-come, first-serve basis.

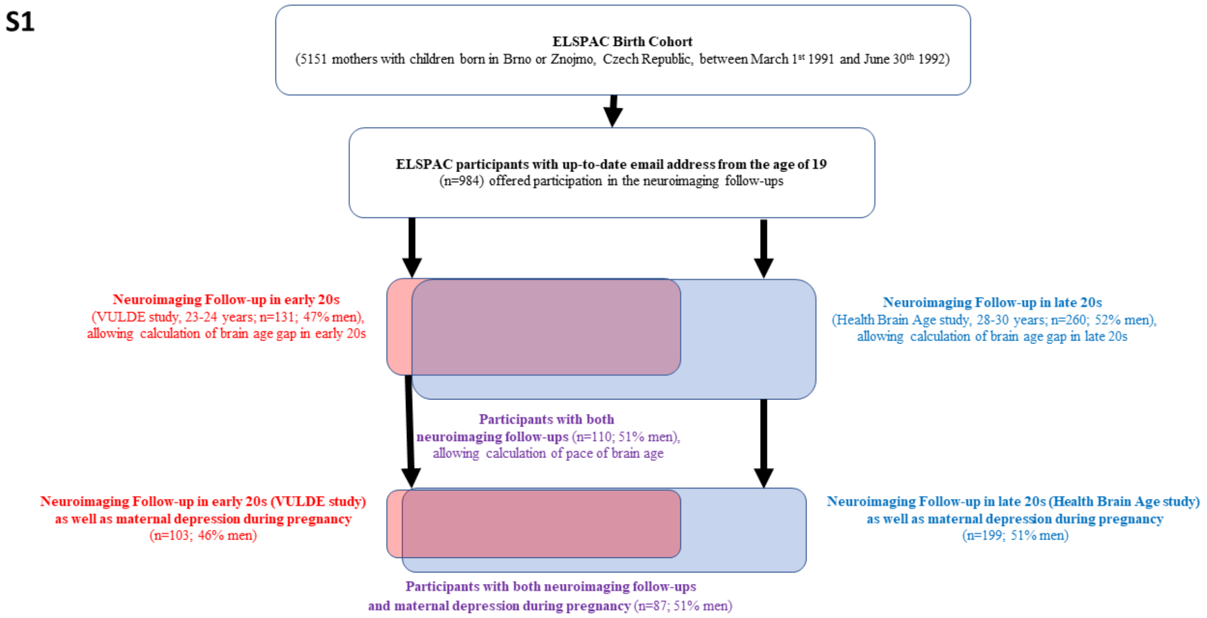

Acquisition of the structural MRI data

During both neuroimaging follow-ups, T1-weighted images were acquired on the same 3T Siemens Prisma MRI scanner with a 64-channel head/neck coil using the same acquisition parameters: voxel size 1x1x1 mm, 240 slices per slab, repetition time (TR) 2300 ms, echo time (TE) 2.34 ms, inversion time (TI) 900 ms, and flip angle 8 degrees

© 2023 Mareckova K et al. *JAMA Network Open.*

eTable 1. Demographics Table

	**Sample A: Health Brain Age MRI** (n=260)	**Sample B: both Health Brain Age and VULDE MRI** (n=110)	**Group difference**
**Sex**<br>% Men	52%	51%	No difference.
**Ethnicity**<br>% White Caucasian	100%	100%	No difference.
**Age at Health Brain Age**<br>in years (M, SD)	M=29.50, SD=0.64	M=29.32, SD=0.61	Sample A > Sample B
**BMI at Health Brain Age** (M, SD)	M=24.26, SD=4.03	M=23.12, SD=3.39	No difference.
**Education**<br>% completed elementary school<br>% completed high school<br>% completed an undergraduate degree<br>% completed a masters degree<br>% completed PhD	1.9%<br>24%<br>10.3%<br>62.3%<br>1.5%	1.8%<br>26.4%<br>8.2%<br>62.7%<br>0.9%	No difference.
**Anxiety trait STAI-T** (M, SD)	M=30.69, SD=0.59	M=30.14, SD=9.34	No difference.
**Depressive symptoms BDI** (M, SD)	M=5.98, SD=5.80	M=6.08, SD=5.30	No difference.
**Stressful life events SSRS** (M, SD)	M=214.32, SD=110.61	M=209.12, SD=113.93	No difference.
**Birthweight**<br>in grams (M, SD)	M=3315.86, SD=501.97	M=3281.94, SD=479.76	No difference.
**Maternal Education**<br>% not completed high school<br>% completed high school<br>% completed university<br>% completed postgraduate educ.	20.1%<br>44.2%<br>32.7%<br>3%	17.9%<br>39.3%<br>39.9%<br>3.6%	No difference.
**Maternal Smoking**<br>% not smoking during pregnancy<br>% smoking during pregnancy<br>% missing	66.4%<br>8.9%<br>23.7%	64.6%<br>12.7%<br>22.7%	No difference.

© 2023 Mareckova K et al. *JAMA Network Open.*

eTable 2. Medication during pregnancy

During this pregnancy, did you take medication in any form (drops, pills, tablets, ointments) for the following problems?	yes, in the first 3 months		yes, later in pregnancy		no, did not use		Missing	
Nausea	27	*10%*	2	*1%*	165	*63%*	68	*26%*
Heartburn	6	*2%*	10	*4%*	175	*67%*	71	*27%*
Vomiting	16	*6%*	3	*1%*	175	*67%*	68	*26%*
Feelings of anxiety	1	*0%*	3	*1%*	191	*73%*	67	*26%*
Infection	26	*10%*	7	*3%*	157	*60%*	72	*27%*
Migraine	4	*2%*	1	*0%*	187	*71%*	70	*27%*
Trouble falling asleep	3	*1%*	2	*1%*	189	*72%*	68	*26%*
Pain	12	*5%*	7	*3%*	176	*67%*	67	*26%*
Allergies	1	*0%*	1	*0%*	192	*73%*	68	*26%*
Skin problems	13	*5%*	3	*1%*	177	*68%*	69	*26%*
Bleeding	13	*5%*	4	*2%*	178	*68%*	67	*26%*
Depression	0	*0%*	1	*0%*	194	*74%*	67	*26%*
Hemorrhoids (golden vein)	4	*2%*	1	*0%*	190	*73%*	67	*26%*
Constipation	8	*3%*	2	*1%*	184	*70%*	68	*26%*
Cough	21	*8%*	6	*2%*	167	*64%*	68	*26%*
Average		*4%*		*1%*		*69%*		*26%*

Did you take any of these medicines during this pregnancy?	yes		no		Missing	
Iron	30	*11%*	160	*61%*	72	*27%*
Zinc	3	*1%*	183	*70%*	76	*29%*
Calcium	32	*12%*	158	*60%*	72	*27%*
Folic acid	19	*7%*	168	*64%*	75	*29%*
Vitamins	112	*43%*	87	*33%*	63	*24%*
Average		*15%*		*58%*		*27%*

Please indicate how often you used the following medicines during pregnancy:	every day		sometimes		never		Missing	
Aspirin (Acylpyrin, Superpyrin, Anopyrin, Acylcofin and others)	2	*1%*	24	*9%*	174	*66%*	62	*24%*
Paracetamol (Paralen, Ataralgin, etc.)	0	*0%*	17	*6%*	181	*69%*	64	*24%*
Codeine (possibly other cough medicines)	0	*0%*	17	*6%*	181	*69%*	64	*24%*
Sleeping pills	0	*0%*	1	*0%*	198	*76%*	63	*24%*
Tranquilizers	0	*0%*	5	*2%*	192	*73%*	65	*25%*
Average		*0%*		*5%*		*71%*		*24%*

© 2023 Mareckova K et al. *JAMA Network Open.*

eTable 3. Correlation matrix describing the relationships between maternal depression measured during the early life of the offspring

Variable	by Variable	Correlation	p-value
Mid-pregnancy	After birth	0.360	<0.0001
Mid-pregnancy	6 months after birth	0.239	0.0009
Mid-pregnancy	18 months after birth	0.276	0.0002
After birth	6 months after birth	0.584	<0.0001
After birth	18 months after birth	0.471	<0.0001
6 months after birth	18 months after birth	0.578	<0.0001

© 2023 Mareckova K et al. *JAMA Network Open.*

eTable 4. Loadings of the main factor describing maternal depression during the early life of the offspring

Assessment of maternal depression	Factor 1 loadings
Mid-pregnancy	0.178
After birth	0.390
6 months after birth	0.751
18 months after birth	0.621

This control analysis showed no significant relationship between perinatal maternal depression and the BrainAGE in the late 20s (beta=0.09, p=0.23) or the pace of aging (beta=0.11, p=0.37).

© 2023 Mareckova K et al. *JAMA Network Open.*

eTable 5. Loadings of the main factor describing anxiety and dysregulated mood in offspring in their late 20s

The measure of anxiety and dysregulated mood	Factor 1 loadings
STAI-T	0.691
STAI-S	0.677
Tension/Anxiety	0.779
Depression/Dejection	0.776
Fatigue/Inertia	0.487
Confusion/Bewilderment	0.687
Anger/Hostility	0.678
Vigor/Activity	-0.215

There was neither a quadratic nor a linear relationship between the general anxiety and mood dysregulation in the late 20s and the BrainAGE in the late 20s (corrected for sex) or the pace of aging in young adulthood (all $p>0.17$). Thus, in contrast to the expected associations between greater BrainAGE and worse anxiety and dysregulated mood, we found no relationship between the BrainAGE in the late 20s or the pace of aging in young adulthood and the general anxiety and dysregulated mood. The absence of a relationship between the general anxiety and dysregulated mood might be related to the relatively low symptoms of anxiety and depression in our sample in the late 20s (82% minimal, 14% mild, 3% borderline and only 1% severe depression, based on the standardized cut-offs for BDI (5)).

© 2023 Mareckova K et al. *JAMA Network Open.*

eResults.

Prevalence of low, moderate and high levels of recent stress

Exposure to recent stress varied from 0 to 561 (M=214.32, SD=110.62). Based on Holmes and Rahe (1) cut-offs for low, moderate, and high levels of stress associated with low, 50% and 80% chance of developing a stress-related disorder, respectively, there was 30% of participants exposed to low levels of stress, 48% participants exposed to moderate levels of stress, 22% of participants exposed to high levels of stress.

Perinatal maternal depression, BrainAGE in the late 20s and the pace of aging

As a control analysis, we also assessed the relationships between perinatal maternal depression, BrainAGE in the late 20s, and the pace of aging. Depression during pregnancy was correlated with the additional postnatal measures of depression (r = 0.24–0.36, see **Supplementary Table 3**), suggesting it may be difficult to disentangle the effects of prenatal maternal depression, which was our main measure of interest and any additional postnatal maternal depressive symptoms on offspring outcomes. Therefore, to demonstrate the specificity of our results, while also taking into account potential multicollinearity concerns, we opted not to include multiple measures of maternal depression in the same model. Instead, similarly to Mareckova et al (2), we conducted a factor analysis using a maximum likelihood factoring method and varimax rotation on all maternal depressive symptom variables (n = 4; maternal depression in mid-pregnancy, after birth, 6 months after birth, and 18 months after birth) and identified a perinatal maternal depression factor (see **Supplementary Table 4** for factor loadings). This factor accounted for 28% of all variance and had significant loadings from all four measures. This factor was used in a control analysis testing the effect of perinatal maternal depression on the BrainAGE in the late 20s and the pace of aging.

BrainAGE in the late 20s, the pace of aging and general anxiety and mood dysregulation in the late 20s

For consistency with our previous work (2), we also assessed the relationships between BrainAGE in the late 20s, the pace of aging and general anxiety and mood dysregulation in the late 20s. Mood dysregulation was assessed using Profile of Mood States (POMS; (3)), a self-report questionnaire assessing current depression/dejection (DD), tension/anxiety (TA), fatigue/inertia (FI), anger/hostility (AH), confusion/bewilderment (CB), and vigor/activity (VA). Anxiety was assessed using Spielberger State-Trait Anxiety Inventory (STAI; (4)), which includes 20 questions assessing anxiety state (STAI-S) and 20 questions assessing anxiety trait (STAI-T). Factor analysis using a maximum likelihood factoring method and varimax rotation across all POMS (DD, TA, FI, AH, CB, VA) and STAI (STAI-S and STAI-T) subscales identified a general mood and anxiety factor capturing 42% of the variance across all measurements. This factor loaded significantly on all subscales except POMS VA and POMS FI (see **Supplementary Table 5** for factor loadings), thus representing general anxiety and mood dysregulation in the offspring in their late 20s. This factor was used to assess the possibly quadratic relationships between (1) the BrainAGE in the late 20s and the general anxiety and mood dysregulation in the late 20s and (2) the pace of aging in young adulthood and the general anxiety and mood dysregulation in the late 20s. Consistent with the guidelines for higher-order polynomial regression analysis, when this higher-order term was insignificant, we repeated the relevant analysis, testing linear effects only.

© 2023 Mareckova K et al. *JAMA Network Open.*

eReferences

1. Holmes TH, Rahe RH. The Social Readjustment Rating Scale. J Psychosom Res. 1967;11(2):213-8.
2. Mareckova K, Marecek R, Andryskova L, Brazdil M, Nikolova YS. Maternal Depressive Symptoms During Pregnancy and Brain Age in Young Adult Offspring: Findings from a Prenatal Birth Cohort. Cereb Cortex. 2020;30(7):3991-9.
3. McNair DM, Lorr, M., & Droppleman, L. F. Manual for the Profile of Mood States. San Diego, CA: Educational and Industrial Testing Services; 1971.
4. Spielberger CD, Gorsuch RL, Lushene R, Vagg PR, Jacobs GA. Manual for the State-Trait Anxiety Inventory. Palo Alto, CA: Consulting Psychological Press; 1983.
5. Beck AT, Steer RA, Brown GK. Manual for the Beck Depression Inventory-II. San Antonio, TX: Psychological Corporation; 1996.

© 2023 Mareckova K et al. *JAMA Network Open.*
