# Supplementary material for: Association of Maternal Depression During Pregnancy and Recent Stress With Brain Age Among Adult Offspring
Source: JAMA Netw Open. 2023 Jan 30;6(1):e2254581. doi: 10.1001/jamanetworkopen.2022.54581 (PMC9887495; doi:10.1001/jamanetworkopen.2022.54581)
Supplement: Supplement 2. — Data Sharing Statement [file jamanetwopen-e2254581-s002.pdf]

Data Sharing Statement

Mareckova. Association of Maternal Depression During Pregnancy and Recent Stress With Brain Age Among Adult Offspring. *JAMA Netw Open*. Published January 30, 2023. doi:10.1001/jamanetworkopen.2022.54581

Data
Data available: No

Additional Information
Explanation for why data not available: Data are available from the first author upon reasonable request.
